# Pathogenic Role of the Sphingosine 1-Phosphate (S1P) Pathway in Common Gynecologic Disorders (GDs): A Possible Novel Therapeutic Target

**DOI:** 10.3390/ijms232113538

**Published:** 2022-11-04

**Authors:** Alice Di Paolo, Arianna Vignini, Sonila Alia, Valentina Membrino, Giovanni Delli Carpini, Luca Giannella, Andrea Ciavattini

**Affiliations:** 1 Department of Clinical Sciences, Section of Biochemistry, Biology and Physics, Università Politecnica delle Marche, 60121 Ancona, Italy; 2 Research Center of Health Education and Health Promotion, Università Politecnica delle Marche, 60121 Ancona, Italy; 3 Department of Clinical Sciences, Section of Obstetrics and Gynecology, Università Politecnica delle Marche, 60121 Ancona, Italy

**Keywords:** endometriosis, adenomyosis, uterine fibroids, ovarian cancer, sphingosine 1-phosphate pathway, S1P receptor modulators

## Abstract

Sphingosine 1-phosphate (S1P) is a bioactive sphingolipid, noteworthy for its involvement both in the modulation of various biological processes and in the development of many diseases. S1P signaling can be either pro or anti-inflammatory, and the sphingosine kinase (SphK)–S1P–S1P receptor (S1PR) axis is a factor in accelerating the growth of several cells, including endometriotic cells and fibrosis. Gynecologic disorders, including endometriosis, adenomyosis, and uterine fibroids are characterized by inflammation and fibrosis. S1P signaling and metabolism have been shown to be dysregulated in those disorders and they are likely implicated in their pathogenesis and pathophysiology. Enzymes responsible for inactivating S1P are the most affected by the dysregulation of S1P balanced levels, thus causing accumulation of sphingolipids within these cells and tissues. The present review highlights the past and latest evidence on the role played by the S1P pathways in common gynecologic disorders (GDs). Furthermore, it discusses potential future approaches in the regulation of this signaling pathway that could represent an innovative and promising therapeutical target, also for ovarian cancer treatment.

## 1. Introduction

Inflammation and fibrosis are common features in gynecological pathologies such as endometriosis, adenomyosis, and uterine fibroids.

Sphingosine 1-phosphate (S1P) is a circulating sphingolipid, which originates from sphyngomyelin catabolism [1]. S1P is formed intracellularly by sphingosine kinase (SK); when exported, it acts through G protein-coupled S1P receptors (S1PRs), which mediate the majority of S1P actions. The metabolism of S1P is impaired in endometriosis and adenomyosis, with a significant remodeling of S1PR expression. In fact, S1P has a role in mediating the profibrotic action of transforming growth factor β (TGFβ1) [2] initially formed, at the site of inflammation, via monocytes and lymphocytes. TGFβ1 stimulates a profibrogenic phenotype with the formation of an extracellular matrix by myofibroblasts that are non-muscle contractile cells triggered in response to injury. Epithelial-to-mesenchymal transition (EMT) and resident fibroblast differentiation into myofibroblasts are the mechanisms which have been suggested to justify the presence of myofibroblasts at the endometriotic lesion level [2]. The S1P pathway and metabolism are also dysregulated in uterine fibroids and in cultures of human leiomyoma cells, inducing the expression of fibrotic markers [3]. In this pathology, similar to TGF-β1, activin A, beside its profibrotic role, thanks to a functional crosstalk with S1P, may promote the fibrotic phenotype [4]. Furthermore, S1P is abnormally produced in ovarian cancer patients, and it may promote the migration, consequent invasion, and the growth of cancer cells as well as tumor angiogenesis, all three processes in which S1P is involved [5]. For this reason, S1P could become a likely molecular target for ovarian cancer treatment.

This comprehensive review aims at exploring various and distinctive perspectives of the relationship between S1P and these common gynecologic disorders (GDs), considers the underlying processes and complex interactions, and sheds light on the role of S1P in their clinical consequences. Lastly, we will discuss new and previously established diagnostic and therapeutic consequences tailored to the context of this relationship.

## 2. Sphingosine 1-Phosphate

S1P is a lipid second messenger, formed by the metabolism of sphingomyelin, involved in the regulation of an array of cellular responses, ranging from proliferation, to differentiation, to migration, and cellular survival [6,7]. S1P is generated inside the cells through the ATP-dependent phosphorylation of sphingosine and by the sphingosine kinases (SphK1 and SphK2) isoenzymes [8]. Being isoenzymes, they promote the catalysis of the same reaction, but they differ in their substrate affinity, tissue allocation, and localization inside the cell. While SphK1 is present, under physiological conditions, in the cytoplasm and then, once activated, it is transferred to the plasma membrane. SphK2 is located inside the nucleus. Even though the two isoenzymes show high homology, they have different functions. In fact, SphK1 displays pro-survival actions, whereas SphK2 has a pro-apoptotic purpose [9]. Different enzymes control the intracellular S1P concentration, mainly SphKs (for its production), sphingosine phosphatase (SPP), and sphingosine lyase (SPL) (for its degradation); more importantly, their ratio is accurately regulated [10]. While SPP dephosphorylates and re-transforms S1P into sphingosine, SPL is able to metabolize it to hexadecenal and phosphoethanolamine, which are then used for the de novo synthesis of phosphatidylethanolamine [10]. 

**Figure 1:**
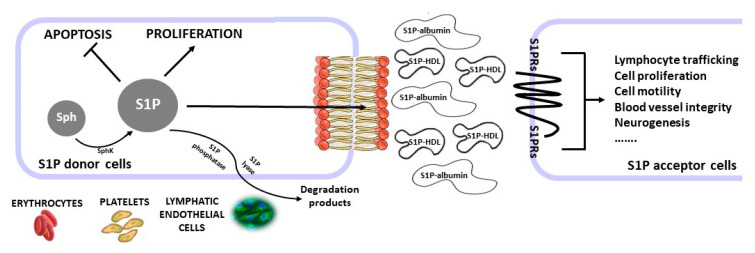
Major signaling pathways and functions of S1P. S1P can act intracellularly to induce either apoptosis or proliferation. S1P can also be exported and can bind S1P receptors in autocrine or paracrine ways. In the circulation, S1P is transported bound to albumin and/or HDL S1P regulates many normal and pathophysiological processes.

After its production, S1P can play its role either as an intracellular messenger, or in paracrine/autocrine pathways, by binding to its membrane receptors 1–5 (S1PR_1–5_). Once exported to plasma or lymph, S1P may propagate the signals [11]. In fact, among all cell types, only blood cells (erythrocytes and platelets) and endothelial cells (vascular and lymphatic) are able to release and export S1P into plasma where, due to their nature, they are bound to proteins (mainly serum albumin) and lipoproteins (primarily high-density lipoproteins) [12]. Human plasma concentrations of S1P average 191–711 nM, whilst on the contrary, tissues and cells comprise only low S1P levels [13]. S1P signaling can be either pro or anti-inflammatory, depending on the background and the tissue source, due to the extensive expression of S1P and S1PRs, which are ubiquitously expressed in different tissues [14]. Growing experimental evidence has shown that S1P is a key molecule that controls different physiological processes, vital for both normal and pathological conditions, including fibrosis (such as fibrosis of lung, heart, skeletal muscles, liver, and kidney), multiple sclerosis (MS), cardiovascular diseases (CVDs) [7], and some forms of cancer [15] (Figure 1). Similarly, the SphKs–S1P–S1PRs pathway plays a role in increased endometriotic cell growth. Mainly, S1P promotes fibrosis in various cells, such as macrophages, fibroblasts [16,17], and skeletal muscle precursors [18], by acting together with other compounds, such as pro-inflammatory cytokines such as tumor necrosis factor α (TNF-α) [19], which in turn stimulate the synthesis of interleukin-1β (IL-1β) and TGF-β in various cells [3]. According to these findings, the regulation of S1P signaling could represent a possible novel therapeutic target [20]. As S1P is implicated in several immune functions, treatments focused on the S1P pathway may also be used to handle autoimmune diseases other than MS [11]. Lastly, therapies targeting S1P and S1P signaling pathways may also be used to treat other different immune-mediated conditions (such as post-stroke or post-infectious) and inflammatory diseases beyond their application in different autoimmune disorders [21].

Given the well-known role of the SphK–S1P signaling axis in many diseases, SphK inhibitors, S1P receptor antagonists, and S1P-blocking antibodies can be used as promising candidates in therapy. In spite of a high level of interest in this field worldwide, few inhibitory compounds have been tested so far. The first FDA-approved S1PR-targeted drug was fingolimod (FTY720) a therapeutic agent in MS. It is an antagonist of S1PR1 expressed on lymphocytes, able to reduce their migration into the CNS, thereby diminishing disease evolution [7]. Fingolimod can cause adverse side effects since it can interact with other S1PRs, different from S1PR1, which are expressed in various tissues, including cardiac myocytes. Nowadays, many clinical trials are in progress to validate the efficacy of the next generation of S1PR-targeted compounds. Siponimod, one of these molecules, also efficacious in MS, is a selective S1PR1 and S1PR5 antagonist, which achieved FDA approval in 2019 [22,23]. At the moment, more studies with longer follow-up are required to evaluate its benefit and to observe long-term undesirable effects.

Concerning the endothelium, S1PR1-biased agonists may have vascular protective actions in dysmetabolic conditions such as diabetes and metabolic and cardiovascular diseases. S1PR1 degradation may cause endothelial positive effects such as NO synthesis, augmented barrier function, and endothelial cell survival, thereby preserving the vasculature [24]. Other S1PRs have been targeted by small antagonists in in vivo and in vitro models of inflammation and fibrosis [25,26].

Taking into account the crucial role of SphK/S1P in cancer evolution, and despite a wealth of literature, to date, no SphK/S1P inhibitor has been employed for cancer therapy. This could be due to the fact that cancer is a multifaceted disease that involves impairments in multiple systems. For this reason, even after the blockade of the SphK–S1P axis, cancer cells may still survive because of other pathways that can evade its inhibition [27,28].

Investigations on sphingolipids and hence its metabolites Sph and S1P have in recent years expanded greatly; therefore, they should be taken into consideration as likely new biomarkers for autoimmune or inflammatory conditions as well as for benign or malignant tumors [29], but more research would benefit from greater attention by researchers and should be implemented.

## 3. S1P Pathway and Common Gynecologic Disorders (GDs)

### 3.1. S1P and Endometriosis

Endometriosis is characterized by the presence of endometrial glands, and stroma in ectopic sites, predominantly the ovaries, pelvic peritoneum, and rectovaginal septum. It affects up to 6–10% of women in their prime reproductive age and it may cause dysmenorrhea, dyspareunia, irregular uterine bleeding, chronic pelvic pain, and/or infertility [30]. Nowadays, endometriosis is under-diagnosed, and it takes a long time for a diagnosis to be made [31]. This pathology is a debilitating condition, causing a significantly low quality of life (QoL) for individuals who are affected by it [32]. The capability of endometrial cells to survive, multiply, and form ectopic endometrial tissues and stroma might be influenced by hormonal factors, altered immunological factors, and genetic factors [33]. Endometriotic tissue’s eutopic endometrium shows a decreased expression of 17-β hydroxysteroid dehydrogenase (17βHSD) type 2 as well as an increased expression of the aromatase enzymes [34]. Hence, a robust increase in the locally bioavailable estradiol level can be considered as a hallmark of the disease. Prostaglandin E2 production is stimulated by estradiol, and this leads to the stimulation of aromatase activity, thus providing a promising therapeutic target [35]. Furthermore, evidence has highlighted a profile of progesterone resistance along with estrogen dependence in the physiopathology of endometriosis [36,37]. Endometriotic lesions showed an overall decrease in progesterone receptor expression, dependent on eutopic endometrium, and an absence of progesterone receptor-β [38]. In the luteal phase, a dysregulation of progesterone responsive genesis is noticeable when analyzing endometrial expression [39,40]. A not complete shift of the endometrium from the proliferative to secretory phase has substantial molecular implications for improving the survival and implantation of refluxed endometrium [41]. Mechanisms such as angiogenesis, inosculation, and vasculogenesis may contribute to the vascularization of endometriotic lesions [42]. Hypoxia is mainly responsible for the genesis of a new microvascular network, which is modulated by several pro- and anti-angiogenic factors [43]. Among them, S1P, acting as a pro-angiogenic factor, seems to play a major role in vascular growth thanks to the activation of SPHK1 by different stimuli such as VEGF, which induces endothelial cell growth [44]. S1P is highly concentrated in the peritoneal cavity, and it increases during menstruation because of menstrual blood reflux. Actually, S1P, derived from erythrocytes during embryogenesis, is essential for the growth of the vascular system and then in the postnatal period, both erythrocytes and the endothelium release S1P into the blood stream in order to preserve vascular homeostasis [45]. 

As endometriosis is recognized as an inflammatory pathology, and growing evidence has suggested that S1P is implicated in inflammatory diseases, the role of the S1P system has been investigated in the progress of endometriosis. For this purpose, studies by Rudzitis-Auth et al., showed that the S1P/SphK1 signaling pathway is implicated in the pathogenesis of endometriosis, as it promotes the establishment and progression of endometriotic lesions [46]. In fact, to achieve their scope, the authors used a recognized mouse model of endometriosis and by the use of SphK1-5C, the specific SphK1 inhibitor, the growth and vascularization of endometriotic lesions was prevented [46].

In accordance with these results, Yoshino et al. in vivo demonstrated that the SphK–S1P–S1PR axis takes part in accelerating the inflammation and growth of endometriotic cells [47] basing their experiments on human cystic fluid of ovarian cysts/tumors and human endometriotic stromal cells (ESC) derived from endometrioma. S1P levels in the cystic fluid of endometriomas appeared to be significantly greater compared to its concentration in nonendometriomas. The same authors also showed that a high amount of S1P (125 nM) was able to increase the ESC cell number by 20%, whereas a low amount of S1P (1.25 nM and 12.5 nM) was able to induce IL-6 mRNA production and IL-6 secretion by ESC, dose-dependently [47]. Furthermore, Bernacchioni et al., through studies on an epithelial-to-mesenchymal transition (EMT) model of uterine adenocarcinoma cells, demonstrated that the S1P signaling axis is profoundly altered in endometriosis. In addition, they showed that S1P is able to mediate the capacity of TGFβ1 to promote fibrosis and EMT markers [2]. 

These data support the assumption that chronic inflammatory conditions, such as in endometriosis, correlated with increased levels of TGF-β, and related growth factors, could alter S1P metabolism and signaling, thus underlining a possible implication of the bioactive sphingolipid in the pathogenesis, as well as in the pathophysiology, of the disease. Therefore, in this context, the S1P pathway may be considered a powerful biomarker for endometriosis and beneficial for diagnostic and therapeutic purposes [2]. Furthermore, experiments by Ono et al. demonstrated that S1P stimulation in human intra-peritoneal macrophages (MΦ) determined an increased expression of IL-6 and COX-2, also causing an extension in the lesion size with CD206+ M2 MΦ. M2 MΦ has already been proven to contribute to angiogenesis through the production of TGF-β1, thus leading to the worsening of endometriosis. The excessive presence of COX-2 expressing MΦ, in patients with endometriosis, might prevent the deletion of refluxed menstrual blood, which causes high S1P levels during the non-menstrual phase [48]. Taken all together, these results revealed that MΦ, in patients with endometriosis, could be stuck in a “vicious cycle” since the phagocytosis of MΦ is decreased because of the increased expression of COX-2. However, the overexpression of COX-2 is caused by S1P stimulation and elevated S1P levels, in the peritoneal cavity, are attributable to the decreased phagocytosis. Therefore, breaking up this cycle (i.e., by reducing S1P levels via inhibition/neutralization of SK) could be a potential therapeutic target. Meanwhile, a confirmatory in vivo study indicated that administration of S1P increased the dimension of the endometriotic-like lesion in a mouse model of endometriosis [48]. 

S1P is also known to stimulate the proliferation of endometriotic cells through the overexpression of the pro-inflammatory cytokine IL-6, related to the formation of endometriotic lesions [49]. Moreover, in endometriotic lesions, the expression of various enzymes, such as SphKs, SPP able to dephosphorylate the compound, and SPL able to definitively degrade it, involved in the cellular conservation of balanced S1P amounts, are shown to be dysregulated [50] with a concomitant accumulation of sphingolipids within the cells. Since S1P operates as a chemoattractant for endothelial cells through the S1P1 receptor, by guiding cells out of tissues, where S1P concentration is relatively low, into circulatory fluids, where its concentration is elevated, it stimulates cell proliferation as well as cell migration [51]. 

Currently, the emerging biological understanding, both on humans and on animal models, is under evaluation in order to assess how to target the SphK1/S1P/S1PRs signal, since S1P was found to possess a very short half-life, ranging from 1 to 15 min according to the studies [52,53]. These data indicate that while S1P is quickly eliminated from circulation by degradative enzymes, it is just as rapidly formed to maintain its high plasma concentration.

The opportunities for therapeutic application comprise antagonists, S1P transporter modulators, compounds able to inactivate key enzymes involved in S1P biosynthesis, biased receptor agonists, and ligand neutralization molecules. Nonetheless, these encouraging candidates for endometriosis treatment have to be proven to be safe and to have an acceptable range of side effects [7].

### 3.2. S1P and Adenomyosis

Adenomyosis is another benign gynecologic condition that, contrariwise to endometriosis, is identified by ectopic endometrial tissue within the uterine myometrium. Women suffering from adenomyosis may have abnormal uterine bleeding (AUB), dysmenorrhea, dyspareunia, or infertility, but one third of them are still asymptomatic [54]. For this reason, the disease prevalence is unclear, though recent data suggest a prevalence ranging from 20% to 35% [55]. For its management, so far there are no international guidelines to follow, and this is of extreme importance as the disease requires a lifelong management plan, including bleeding and pain control, fertility maintenance, and pregnancy outcome [56]. Adenomyosis often co-occurs with other gynecological pathologies, such as endometriosis and uterine fibroids [57].

To date, the pathogenic mechanisms involved in adenomyosis need to be fully elucidated, but in the last few years, numerous studies have shown that sex steroid hormone receptors, inflammatory molecules, extracellular matrix enzymes, growth, and neuroangiogenic factors are fundamental in its development [54]. As described for endometriosis, also in adenomyosis the S1P signaling axis shows profound dysregulation. So far, only one paper has been published on this issue showing that the S1P bioactive lipid could be implicated in the fibrotic signature typical of the disease [58], with an increased expression of S1P receptor S1P_3_, and a lower expression of mRNA levels, when compared with a healthy endometrium. In addition, the same authors demonstrated a correlation with actin-alpha-2 smooth muscle (coded by ACTA2 gene) expression, a gene required in fibrogenesis [58]. These preliminary data suggest that the S1P pathway could be a possible innovative target for future adenomyosis therapy.

### 3.3. S1P and Uterine Fibroids 

Uterine leiomyomas, commonly known as uterine fibroids, are the most typical benign tumor of the uterus, affecting reproductive-age women, with a prevalence estimated at 4.5–68.6% [59]. Irregular and excessive menstrual bleeding, with secondary anemia, pelvic pain, and pressure are recurrent symptoms associated with uterine leiomyomas; an increase in obstetric complications is also described in affected women. Uterine fibroids stem from the myometrial smooth muscle cells of the uterus and are delineated by excessive deposition of extracellular matrix (ECM) proteins, including collagens, fibronectin, and proteoglycans that identify fibrosis, as well as very high levels of inflammatory mediators such as cytokines and chemokines [60,61]; thereby, they are also defined as fibrotic disease [62,63]. 

An uncommon myofibroblast and stem cell activation, along with an irregular fibrinogenesis and inflammatory response, may determine uterine fibroids [64]. Progenitor cells (PCs) obtained from uterine leiomyomas and normal myometrium were demonstrated to secrete different amounts of cytokines implicated in acute and chronic inflammation [65]; specifically, upregulation of the latter cytokines evince an imbalance that could stimulate a microenvironment suitable for uterine leiomyoma onset and growth. In a recent study, Lazzarini et al. supported the hypothesis that fibroids result from changes occurring in PCs [66], as only 15 out of 2646 microRNAs (miRNAs) are differentially regulated in the normal myometrium and leiomyoma and they are implicated in seven dysregulated pathways. Symptoms such as abnormal bleeding, pelvic pressure, and pain are due to an overproduction of ECM that causes rigidity of the structure [67,68].

Accumulating evidence has indicated that Activin A, a new protein with features in wound repair, fibrosis, cell proliferation, differentiation, apoptosis, and metabolism, is one of the TGF-β that is overexpressed in uterine fibroids, and it also participates in critical pathologic processes that contribute to fibrosis [69]. Activin A plays a major role in the induction of fibrotic phenotypes in uterine fibroid cells [70]. 

To reinforce these findings, Bernacchioni et al. [3] demonstrated that SphK1 and SphK2 mRNA levels were significantly higher in uterine fibroids than in adjacent healthy endometrial tissues. Furthermore, they showed that activin A mRNA levels were higher in uterine fibroids compared to adjacent myometrial explants. Similarly, S1PR2, S1PR3, and S1PR5 mRNA levels were significantly higher in uterine fibroids in comparison with myometrial healthy tissues; both S1PR2 and S1PR3 protein levels were also elevated in the leiomyoma. These results suggest that S1P signal dysregulation is detectable in uterine fibroids [3]. Moreover, the fibrotic roles of S1P signaling were tested in uterine fibroids and myometrial cells. The results indicated that S1P significantly enhanced mRNA expression of ECM proteins, collagen, and fibronectin in fibroid cells, but not in myometrial cells [3]. Lastly, S1P was also able to increase mRNA expression of profibrotic growth factor activin A in fibroid cells, at least in part, by overexpressing activin A [3].

Indeed, some therapies for the management of fibroid-related symptoms have been approved but they are not free from side effects, and they have not been approved for long-term use in some patients. As mentioned previously, inhibitors of the SphK/S1P/SP1R signaling pathway are effective in many human and animal models, and the results herein summarized show a role of S1P and its signaling axis in fibrotic process of leiomyoma and suggest its potential role as target of fibromatosis and therapy [3,4].

### 3.4. S1P and Other Gynecological Diseases 

Growing evidence also supports the important role of S1P in reproductive functions and fertility. S1P and other phospholipid-derived mediators seem to play a role in endometrial receptiveness, embryonic spacing, and decidualization based on animal and human studies.

Regarding ovarian cells, S1P synthesis is involved in follicular health [71], in ovulation and the development of corpus luteum [72], and the synthesis of sexual steroids [73]. Hexogen S1P administration seems to protect ovarian cells from either irradiation or cytotoxic drugs used to treat cancer [74], albeit the deletion of SIP receptors (i.e., S1PR2 or S1PR3) negatively affects fertility [75], suggesting a pivotal role in follicular function preservation. S1P appears to be an essential stimulator in the preantral and antral phase of follicular development, as well as during ovulation and corpus luteum development. Lack of S1P synthesis, as well as its overproduction, may be implicated in ovarian hyperstimulation syndrome (OHSS) [5].

Bioactive sphingolipids are also known to play an important role in the development, progression, and metastasization of cancer; in particular, S1P is able to regulate cell growth [76] and cell trafficking [11] and suppresses apoptosis [77]; thereby, it might play a pivotal role in cancer and gynecological neoplasia. Overexpression of SphK1 is effective in promotion, while its inhibition reduces tumor growth, angiogenesis, and chemoresistance in various xenograft models [1]. Going into more detail, the S1P/SphK1 pathway is involved in the main mechanisms favoring oncogenesis, as well as promoting cell survival, proliferation, and transformation, apoptosis prevention, and angiogenesis stimulation [78]. Regarding endometrial carcinoma, Knapp et al. [78] showed that it is characterized by profound changes in the sphingolipid metabolism that might contribute to its progression and chemoresistance. Sphingolipid metabolism in the human endometrial carcinoma is significantly up-regulated in comparison with a healthy endometrium. High levels of S1P are found in endometrial carcinoma, resulting from both the activation of SphK1 and the abundance of its substrate, sphingosine, and there is evidence that SphK1 inhibitors reduce cell proliferation and tumor growth both in vitro and in vivo [79]. From experiments by Dai et al. [80], we became aware that SphK1/S1P/S1PR1/3 signaling plays an important role in ovarian cancer angiogenesis and the stoppage of this pathway could significantly inhibit the process. It has already been shown that levels of SphK1 are significantly increased in ovarian cancer tissue [81]. In addition, plasma S1P levels were found to be elevated in patients with ovarian cancer and decreased following removal of the tumor [82]. Moreover, SphK1, but not SphK2, expression levels were demonstrated to be correlated with microvascular density (MVD) of ovarian cancer tissue; the angiogenic potential and the angiogenic factor secretion of ovarian cancer cells could be mitigated by SphK1 blockage and were shown to be restored by adding S1P [80]. Indeed, S1P induced the angiogenic factor expression via S1PR1 and S1PR3 in ovarian cancer cells; in fact, blocking SphK or S1PR1/3 might functionally inhibit ovarian cancer angiogenesis. It can thus be assumed that either S1P or S1PR1/3 are responsible for the production of the angiogenic factor and the angiogenic potential of ovarian cancer: S1P and S1PR1/3 antagonists (i.e., SphKI-II and VPC23019, respectively) could block, or at least attenuate, the pro-angiogenic effects of both of them [80].

## 4. Conclusions

S1P is a bioactive sphingolipid metabolite that plays a crucial role in modulating various biological processes, as well as in developing many diseases, in particular those related to fibrosis and inflammation, conditions that characterize the onset of gynecological diseases such as endometriosis, adenomyosis, and uterine fibroids [7]. Most of the studies, present in the literature, have underlined a possible pathogenic role of the dysregulation of S1P levels in endometriotic disease, through a direct or indirect modulation of fibrosis and activation of chronic inflammatory processes which may involve TGF-β [2,50], and finally induce proliferation of endometriotic cells and lesion growth [47]. Therefore, the S1P signaling axis could represent an interesting and powerful biomarker for endometriosis and a useful target for diagnostic and therapeutic purposes. As for endometriosis, also in adenomyosis and in uterine fibroids, the S1P signaling axis shows a deep dysregulation [3]. An altered S1P signaling pathway might be implicated in the fibrotic phenotype of adenomyosis and a correlation with actin-alpha-2 smooth expression has been evinced [58]. Elevated mRNA levels of SphK1, SphK2, activin A, S1PR2, S1PR3, and S1PR5 are present in uterine fibroids in comparison with healthy controls, as well as increased S1PR2 and S1PR3 protein levels [3]. An altered SphK1/S1P/S1PR1/3 signaling axis could play a crucial role in ovarian cancer angiogenesis, although further explorations on intracellular S1P functions should be conducted, keeping in mind its biological action exerted due to S1PR binding [14,83]. Regulation of the S1P pathway may represent a potential new therapeutic target, as S1P seems to exert cytoprotective consequences against cancer treatment side effects (i.e., chemotherapy) [5]. 

All the in vitro and in vivo studies since S1P was first discovered as a second messenger have shown us much about its mechanisms of action. We are now aware why S1P is so important for the regulation of many normal and pathophysiological processes, such as autoimmune or inflammatory disorders, as well as benign or malignant tumors, but further investigation is needed since its physiologic roles are not yet fully understood. Despite this, the discovery of S1P intracellular targets will offer a wide range of research opportunities in order to ascertain the role of S1P as an anti or pro-inflammatory signaling compound and to translate this information into a novel class of sphingolipid-centric therapeutics.

## Data Availability

No new data were created or analyzed in this study. Data sharing is not applicable to this article.
